# Maternal Hyperglycemia Promotes Late-Life Neurodegenerative Changes Associated with AGE–RAGE Activation and Impaired Insulin Signaling: Protective Effects of Palmitoleic Acid

**DOI:** 10.3390/nu18111748

**Published:** 2026-05-29

**Authors:** Ritsuko Kawaharada, Reiko Kimura, Eri Miyata, Reona Noguchi, Akiyo Toriumi, Akio Nakamura

**Affiliations:** 1 Department of Food and Nutrition, Takasaki University Graduate School of Health and Welfare, 37-1, Nakaorui-machi, Takasaki 370-0033, Gunma, Japan; nasu@takasaki-u.ac.jp (R.K.); 2610301@takasaki-u.ac.jp (R.K.); 2 Department of General Surgical Science, Graduate School of Medicine, Gunma University, Maebashi 371-8511, Gunma, Japan; m2510005@gunma-u.ac.jp; 3 Department of Nutrition, Takasaki University of Health and Welfare, 37-1, Nakaorui-machi, Takasaki 370-0033, Gunma, Japan; noguchi-r@takasaki-u.ac.jp; 4 Department of Public Health, Graduate School of Medicine, Gunma University, Maebashi 371-8511, Gunma, Japan; m2320028@gunma-u.ac.jp; 5 Department of Molecular Nutrition, Graduate School of Human Life Sciences, Jissen Women’s University, 4-1-1, Osakaue, Hino 191-8510, Tokyo, Japan

**Keywords:** gestational diabetes mellitus, neurodegeneration, AGE–RAGE signaling, insulin signaling, oxidative stress, cognitive impairment, palmitoleic acid

## Abstract

**Background/Objectives:** Maternal hyperglycemia is associated with adverse neurodevelopmental outcomes in offspring; however, its long-term effects on brain aging remain unclear. This study investigated whether maternal hyperglycemia induces persistent molecular and behavioral alterations in aged male offspring and whether maternal palmitoleic acid supplementation exerts protective effects. **Methods:** The pregnant rats were divided into four groups: PCM, PDM, PDM/CPA, and PDM/TPA. Male offspring were analyzed at 48 weeks of age. **Results:** Maternal hyperglycemia significantly increased cerebral RAGE expression (~1.6-fold) and tau phosphorylation (~1.8-fold), accompanied by reduced Akt phosphorylation, impaired Nrf2-related antioxidant responses, and increased inflammatory gene expression. These molecular alterations are associated with impaired recognition memory, as reflected by a marked reduction in the discrimination index in the novel object recognition test. Maternal CPA/TPA supplementation partially attenuated these abnormalities. **Conclusions:** These findings suggest that maternal hyperglycemia may induce long-lasting molecular alterations associated with neuroinflammation, impaired insulin-related signaling, and cognitive dysfunction in aged offspring. Maternal palmitoleic acid supplementation may partially mitigate the adverse developmental alterations associated with intrauterine hyperglycemic exposure.

## 1. Introduction

Gestational diabetes mellitus (GDM) is a form of glucose intolerance that is first recognized or diagnosed during pregnancy, and its prevalence has been increasing worldwide in recent years. GDM affects maternal metabolism and the long-term health of the offspring. Exposure to a hyperglycemic intrauterine environment has been suggested to influence postnatal metabolic abnormalities and neurodevelopment. Epidemiological studies have reported that children born to mothers with diabetes are at an increased risk of obesity and type 2 diabetes and neurodevelopmental disorders such as attention-deficit/hyperactivity disorder and autism spectrum disorder [1,2,3]. These findings strongly support the concept of Developmental Origins of Health and Disease, suggesting that the intrauterine metabolic environment influences postnatal health outcomes [4].

Therefore, several studies have been actively conducted using animal models of gestational diabetes. In offspring of streptozotocin-induced gestational diabetic rats, reduced neuronal density, impaired synapse formation, and decreased neural plasticity have been reported in the hippocampus and cerebral cortex [5,6,7]. These structural and molecular alterations may impair neural circuit formation and synaptic connectivity, thereby contributing to cognitive dysfunction [8,9].

Furthermore, abnormalities in cognitive and emotional behaviors have been reported in offspring of gestational diabetes models. For example, impaired recognition memory in the novel object recognition test and deficits in spatial learning in the water maze test have been observed [9,10]. Additionally, anxiety-like behavior and altered exploratory activity have been reported in the elevated plus maze and open field tests [9,11]. These findings suggest that fetal exposure to hyperglycemia exerts long-term effects on neural circuit formation and brain function [8,9].

The involvement of glycation stress and neuroinflammation has recently attracted attention as a potential mechanism underlying the effects of gestational diabetes on the fetal brain. Under hyperglycemic conditions, the formation of advanced glycation end products (AGEs) is accelerated, and AGEs activate inflammatory signaling by binding to their receptor, receptor of AGE (RAGE) [12]. The AGE–RAGE signaling pathway induces inflammatory cytokine production and oxidative stress via the nuclear factor kappa B (NF-κB) pathway [13]. Increased expression of glial fibrillary acidic protein (GFAP) and ionized calcium-binding adapter molecule 1 (Iba1) has been reported in the brains of offspring in animal models of gestational diabetes, suggesting enhanced neuroinflammation [10,14]. Furthermore, abnormalities in the insulin signaling pathways and alterations in synapse-related proteins have been reported, indicating that the fetal metabolic environment may influence neural function [15,16]. Accumulating evidence suggests that chronic neuroinflammation, oxidative stress, and insulin signaling dysfunction are closely associated with brain aging and neurodegenerative disorders, including Alzheimer’s disease [17,18,19]. Persistent AGE–RAGE signaling has been implicated in age-related cognitive decline by promoting oxidative stress, microglial activation, and synaptic dysfunction [20,21]. These findings raise the possibility that fetal exposure to hyperglycemia may increase the susceptibility to age-associated neurological dysfunction later in life.

We have previously reported enhanced AGE–RAGE signaling and impaired insulin-related signaling in the fetal brains of gestational diabetes model rats. Although previous studies have suggested neurodevelopmental abnormalities in offspring exposed to gestational diabetes, most investigations have focused on the fetal, neonatal, or young adult stages. Consequently, it remains unclear whether intrauterine hyperglycemia induces persistent molecular alterations that contribute to brain aging and late-life neurodegenerative vulnerabilities. In particular, the long-term involvement of AGE–RAGE signaling, impaired insulin signaling, oxidative stress responses, and tau-related neurodegenerative pathways in aged offspring has not been sufficiently investigated. Additionally, administration of palmitoleic acids, including cis-palmitoleic acid (CPA) and trans-palmitoleic acid (TPA), ameliorated these molecular abnormalities in pregnant rats [22]. Cis- and trans-palmitoleic acids were examined separately, because they differ in their endogenous origins and dietary sources. Cis-palmitoleic acid is synthesized endogenously from palmitic acid via stearoyl-CoA desaturase in the human body and is abundant in foods such as macadamia nut oil and avocado oil. In contrast, trans-palmitoleic acid is primarily derived from dairy and ruminant fats. Therefore, we analyzed cis- and trans-palmitoleic acids separately to evaluate whether different dietary sources and structural isomers exert distinct biological effects as functional lipids.

We hypothesized that fetal exposure to maternal hyperglycemia induces persistent activation of AGE–RAGE signaling and impairs insulin signaling and Nrf2-dependent antioxidant defense pathways, thereby promoting neuroinflammation, tau phosphorylation, and age-related cognitive dysfunction in the offspring. To test this hypothesis, we evaluated the aged offspring of rats with gestational diabetes to determine whether fetal hyperglycemic exposure promotes molecular and behavioral features associated with brain aging and neurodegenerative vulnerability. Furthermore, we investigated whether maternal administration of palmitoleic acids (CPA and TPA) could attenuate these long-term alterations.

## 2. Materials and Methods

### 2.1. Animals

All animal experiments were approved by the Animal Experiment Committee of Takasaki University of Health and Welfare (approval number: 2508; 37-1 Nakaimachi, Takasaki, Gunma 370-0033, Japan). Pregnant Wistar rats on gestational day 1 were purchased from CLEA Japan Inc. (from Tokyo, Japan). and randomly assigned to either the control group (*n* = 5) or the GDM group (*n* = 15). Experimental GDM was induced by intravenous injection of streptozotocin (streptozotocin; 40 mg/kg; FUJIFILM Wako Pure Chemical Corporation, Osaka, Japan) dissolved in 50 mM citrate buffer (pH 4.5) on gestational day two. Pregnant rats with blood glucose levels exceeding 350 mg/dL at 24 h after streptozotocin injection were considered diabetic and included in the GDM group.

The GDM rats were further divided into three treatment groups receiving oral administration of cis-palmitoleic acid (CPA; *n* = 5, 150 mg/kg/day; Cayman Chemical, Ann Arbor, MI, USA), transpalmitoleic acid (TPA; *n* = 5, 150 mg/kg/day; ABITEC, Larodan Research Grade Lipids, Solna, Sweden), or water (*n* = 5). CPA and TPA doses were determined based on a previous study [22].

Four groups of offspring were established: offspring of control dams (PCM group, *n* = 5), offspring of CPA-treated hyperglycemic dams (PDM/CPA group, *n* = 5), offspring of TPA-treated hyperglycemic dams (PDM/TPA group, *n* = 5), and offspring of hyperglycemic dams (PDM group, *n* = 5). All animals were fed a standard laboratory diet (CE-2; CLEA Japan, Inc., Tokyo, Japan) throughout the experimental period. The composition of the CE-2 diet was 4.8% fat, 49.8% carbohydrates, 25.1% protein, 4.4% fiber, and 8.9% moisture, with a caloric value of approximately 3424 kcal/kg.

Animals were individually housed in plastic cages with wood chip bedding under controlled environmental conditions (60 ± 5% humidity) and maintained on a 12 h light/dark cycle. Only male offspring were used for subsequent analyses to minimize potential confounding effects of sex differences. All offspring were reared under identical conditions and maintained until 48 weeks of age, representing an aged stage in rats. At this point, behavioral assessments and analyses of cerebral tissue were performed. At the conclusion of the study, all rats were euthanized by isoflurane inhalation in accordance with the institutional guidelines.

### 2.2. Western Blot Analysis

Brain tissues from 48-week-old offspring were processed using the NucleoSpin^®^ TriPrep Total DNA, RNA, and Protein Isolation Kit (MACHEREY-NAGEL GmbH & Co. KG, Düren, Germany). Protein pellets were dissolved in the Laemmli sample buffer. Equal amounts of total protein were separated using sodium dodecyl sulfate–polyacrylamide gel electrophoresis and transferred onto polyvinylidene difluoride membranes (Merck Millipore, Tokyo, Japan). The membranes were blocked with EveryBlot blocking buffer (Bio-Rad, Hercules, CA, USA) and incubated overnight at 4 °C with primary antibodies (1:1000). After washing, the membranes were incubated with horseradish peroxidase-conjugated secondary antibodies (1:5000) for 1 h at room temperature.

Protein bands were visualized using the Immobilon Western Chemiluminescent HRP Substrate (Merck Millipore, Tokyo, Japan) and quantified using a Fusion S Imaging Analyzer (Vilber Lourmat, Collégien, France).

The following primary antibodies were used: anti-protein kinase B (Akt) (#9272), anti-phospho-Akt (p-Akt473) (Ser473; #9267), β-tubulin (#2128), anti-Tau (Pan-Tau) (Tau46; #4019), anti-phospho-Tau (p-Tau181) (Thr181; #12885), anti-phospho-Tau (p-Tau231) (Thr231; #71429), anti-phospho-Tau (p-Tau217) (Thr217; #51625), anti-Iba1/AIF-1 (Iba1) (#17198), anti-Glial Fibrillary Acidic Protein (GFAP) (#12389), anti-postsynaptic density protein 95 (PSD-95) (#3450), anti-Synaptophysin (#36406), and anti-NeuN (#24307) (all from Cell Signaling Technology, Danvers, MA, USA); anti-AGEs antibody (KH001) from TransGenic Inc. (from Fukuoka, Japan); and anti-RAGE antibody (ab3611) from Abcam plc (from Cambridge, UK). Horseradish peroxidase-conjugated anti-rabbit IgG (#7074) and anti-mouse IgG (#7076) antibodies were purchased from Cell Signaling Technology (from MA, USA).

### 2.3. RNA Extraction and RT-qPCR

Total RNA was extracted from brain tissues using the NucleoSpin^®^ TriPrep Total DNA, RNA, and Protein Isolation Kit (MACHEREY-NAGEL GmbH & Co. KG, Düren, Germany). Complementary DNA was synthesized using ReverTra Ace^®^ qPCR RT Master Mix with gDNA Remover (TOYOBO Co., Ltd., Osaka, Japan). RT-qPCR was performed using the QuantStudio™ 3 Real-Time PCR System (Thermo Fisher Scientific, Waltham, MA, USA) and KOD SYBR qPCR Mix (TOYOBO Co., Ltd., Osaka, Japan).

PCR conditions were as follows: initial denaturation at 98 °C for 2 min, followed by 40 cycles of 98 °C for 10 s, 60 °C for 10 s, and 68 °C for 30 s. Primer sequences used in this study are listed in Supplementary Table S1. The mRNA expression levels of *Slc6a3* (Solute Carrier Family 6 Member 3), *Drd2* (Dopamine Receptor D2), *Comt* (Catechol-O-Methyltransferase), *Snap25* (Synaptosome Associated Protein 25), *Ntrk2* (Neurotrophic Receptor Tyrosine Kinase 2), *Mecp2* (Methyl-CpG Binding Protein 2), *Nrf2* (NFE2 Like BZIP Transcription Factor 2), *Hmox1* (Heme Oxygenase 1), *Gclm* (Glutamate-Cysteine Ligase Modifier Subunit), *Txnrd1* (Thioredoxin Reductase 1), *Cat* (Catalase), *Sod1* (Superoxide Dismutase 1), *BDNF* (Brain Derived Neurotrophic Factor), *IL-6* (Interleukin 6), *TNF-α* (Tumor Necrosis Factor), *RAGE* (Advanced Glycosylation End-Product Specific Receptor), *Map2 (Microtubule Associated Protein 2)*, *Rbfox3* (RNA Binding Fox-1 Homolog 3) and *Tubb3* (Tubulin Beta 3 Class III) were normalized to the endogenous reference genes *Rplp0* (Ribosomal Protein Lateral Stalk Subunit P0) and *HPRT1 (Hypoxanthine Phosphoribosyltransferase 1)*. Relative expression levels were calculated using the comparative Ct method (2^−ΔΔCt^).

### 2.4. Behavioral Assessment

Behavioral tests were conducted on 48-week-old male offspring rats to evaluate their cognitive and emotional functions. The following tests were performed according to standard protocols.

- Y-maze test (short-term spatial memory)
- Elevated plus maze test (anxiety-like behavior)
- Novel object recognition test (recognition memory)

All behavioral analyses were performed by investigators who were blinded to the treatment groups. Behavioral parameters were recorded by direct observation without an automated video tracking system and manually scored according to predefined behavioral criteria.

#### 2.4.1. Y-Maze Test

The Y-maze test was used to evaluate spatial working memory and exploratory behavior. Each rat was placed at the end of one arm and allowed to explore freely for 8 min. The exploratory activity was defined as the total number of arm entries. Spatial memory performance was evaluated based on spontaneous alternation behavior. Spontaneous alternation was defined as consecutive entries into all three arms. The percentage of spontaneous alternations was calculated as follows:

Spontaneous alternation (%) = [A/(E − 2)] × 100

where A represents the number of alternations and E represents the total number of arm entries.

#### 2.4.2. Elevated Plus Maze Test

The elevated plus maze test was used to assess anxiety-like behavior. The apparatus consisted of two open arms (50 × 10 cm) and two closed arms (50 × 10 cm, with 40 cm-high walls), both elevated 50 cm above the floor. Before testing, rats were habituated to the test room for 30 min. Each animal was placed in the center of the maze and allowed to explore freely for 5 min. The time spent in the open arms and the percentage of open-arm entries were recorded.

#### 2.4.3. Novel Object Recognition Test

A novel object recognition test was conducted using an open-field apparatus (60 cm × 60 cm × 40 cm) to evaluate recognition memory. During the habituation phase, rats were allowed to explore the empty apparatus for 10 min. During the training phase, two identical objects were placed diagonally in the apparatus, and the rats were allowed to explore for 10 min. After 24 h, one familiar object was replaced with a novel object, and the rats were allowed to explore for 5 min.

Exploratory behavior was defined as directing the nose toward, sniffing, or touching an object within 2 cm. Recognition memory was quantified using the discrimination index (DI).

DI = (time spent exploring the novel object − time spent exploring the familiar object)/total exploration time.

### 2.5. Blood Glucose Levels and Body Weight

Blood glucose levels and body weights were measured weekly. Blood glucose levels were measured using the Glutest Neo sensor (SANWA KAGAKU KENKYUSHO CO., LTD., Nagoya, Japan) based on the glucose dehydrogenase-flavin adenine dinucleotide electrode method. Subsequently, 50% glucose solution (3 g/kg) was administered, and blood glucose levels were measured at 0, 30, 60, 90 and 120 min. The area under the curve (AUC) was calculated.

### 2.6. Statistical Analysis

Normality of data distribution was assessed using the Shapiro–Wilk test, and homogeneity of variance was evaluated using Levene’s test before group comparisons. When the assumption of equal variance was satisfied, a one-way ANOVA followed by Tukey’s HSD post hoc test was applied. When the homogeneity of variance was violated, Welch’s ANOVA followed by Games–Howell post hoc testing was used. Statistical significance was defined as *p* < 0.05. Post hoc power analyses for the behavioral experiments were performed using G*Power software (version 3.1, Heinrich Heine University, Düsseldorf, Germany) based on one-way ANOVA models. Statistical power (1 − β) was calculated using the observed effect sizes, sample sizes, and significance level (α = 0.05) obtained from the behavioral data analyses.

## 3. Results

### 3.1. Effects of CPA and TPA on Glucose Tolerance and Body Weight in GDM Rats (Table 1 and Figure 1)

An oral glucose tolerance test (OGTT) was performed to evaluate the effects of maternal hyperglycemia and CPA or TPA intake during pregnancy on offspring glucose tolerance. Analysis of blood glucose levels at 0 min (before glucose loading) and at 30, 60, 90, and 120 min after loading, as well as the area under the OGTT curve, revealed no significant differences between the groups. No differences in body weight were observed between the groups.

**Table 1:** Body weights and blood glucose levels of the rat offspring groups.

	PCM	PDM	PDM/CPA	PDM/TPA
Blood glucose (mg/dL)	90.1 ± 15.2	92.3 ± 13.7	97.7 ± 18.7	95.3 ± 11.1
Body weight (g)	503.7 ± 15.9	503.2 ± 14.8	512.5 ± 18.1	508.5 ± 16.3

Each value represents the mean ± SEM. PDM, offspring of hyperglycemic dams; PCM, offspring of control dams. The rat offspring of hyperglycemic dams fed CPA or TPA were designated as PDM/CPA or PDM/TPA, respectively.

**Figure 1:**
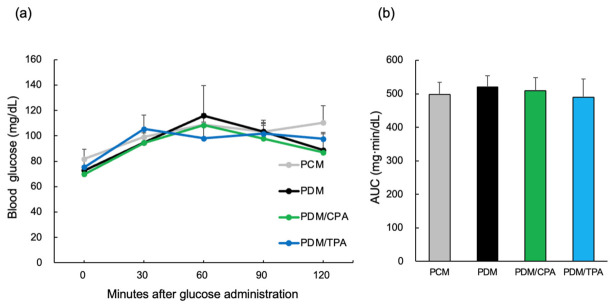
Effects of maternal diabetes and palmitoleic acid supplementation on glucose tolerance in aged offspring rats. An oral glucose tolerance test (OGTT) was performed on 48-week-old male offspring of control dams (PCM), hyperglycemic dams (PDM), and hyperglycemic dams treated with cis- (PDM/CPA) or trans-palmitoleic acid (PDM/TPA) during pregnancy. Following oral glucose administration (3 g/kg), blood glucose levels were measured at the indicated time points. (**a**) Time-course changes in blood glucose levels during the OGTT. (**b**) Area under the curve (AUC) of glucose levels during OGTT. Data are presented as mean ± SEM (*n* = 5 per group).

### 3.2. AGEs–RAGE Signaling and Insulin Signaling in the Brain (Figure 2)

The effects of maternal hyperglycemia on glycation stress and insulin signaling in the cerebrum of adult offspring were evaluated. AGE expression was significantly higher in the PDM group than in the PCM group. Expression of RAGE, an AGE receptor, was also significantly higher in the PDM group than in the PCM group. However, the administration of CPA or TPA decreased RAGE expression compared with the PDM group.

**Figure 2:**
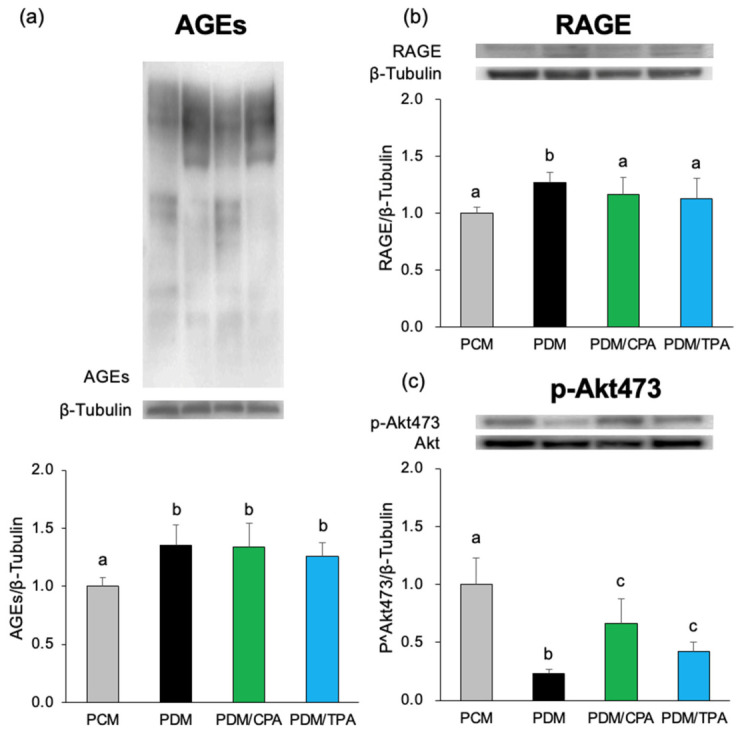
Increased AGE–RAGE signaling and impaired Akt phosphorylation in the brains of aged offspring exposed to maternal hyperglycemia. Western blot analysis was performed using cerebral tissue obtained from 48-week-old male offspring of control dams (PCM), hyperglycemic dams (PDM), and hyperglycemic dams treated with cis-palmitoleic acid (PDM/CPA) or trans-palmitoleic acid (PDM/TPA) during pregnancy. Representative immunoblots of AGEs, RAGE, phosphorylated Akt at Ser473 (p-Akt473), total Akt, and β-tubulin are shown. (**a**) Quantification of AGEs normalized to β-tubulin. (**b**) Quantification of RAGE normalized to β-tubulin. (**c**) Quantification of p-Akt473 normalized to total Akt levels. Data are presented as mean ± SEM (*n* = 5 per group). Statistical analysis was performed using one-way ANOVA, followed by Tukey’s HSD post hoc test. Different letters (a–c) indicate statistically significant differences between groups (*p* < 0.05).

Furthermore, the expression of p-Akt473, an indicator of insulin signaling, was lower in the PDM group than in the PCM group. Phosphorylation of Akt increased in the PDM/CPA and PDM/TPA groups compared with the PDM group, suggesting a tendency toward recovery of insulin signaling.

### 3.3. Neuroinflammation and Synaptic Protein Expression (Figure 3)

The effects of maternal hyperglycemia on neuroinflammation and synapse-related proteins in the brains of offspring were evaluated. The expression of GFAP, an astrocyte marker, was higher in the PDM group than in the PCM group. In contrast, expression appeared to be lower in the PDM/CPA and PDM/TPA groups compared with the PDM group. Expression of Iba1, a microglial marker, was higher in the PDM group than in the PCM group.

**Figure 3:**
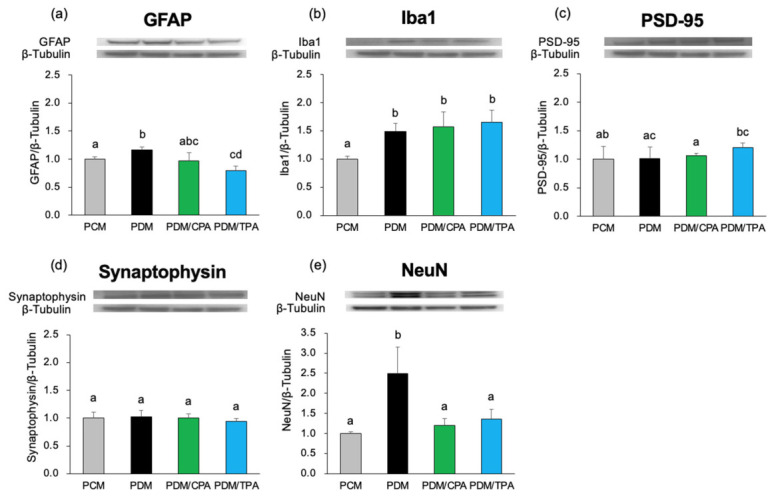
Alterations in neuroinflammation and synaptic proteins in offspring brains. Western blot analysis of neuroinflammatory markers and synaptic proteins in the brain tissue. (**a**–**e**) Representative immunoblots for GFAP, Iba1, NeuN, PSD-95, and synaptophysin. Protein expression was normalized to β-tubulin. Data are presented as mean ± SEM. Statistical analyses were performed using one-way analysis of variance (ANOVA), followed by Tukey’s honest significant difference post hoc test. Different letters (a–d) indicate statistically significant differences between groups (*p* < 0.05).

There were no differences in the expression of PSD-95 and synaptophysin between the PCM and PDM groups. The expression of NeuN, a neuronal marker, was higher in the PDM group than in the PCM group, but lower in the PDM/CPA and PDM/TPA groups than in the PDM group.

### 3.4. Tau Phosphorylation in the Brain (Figure 4)

Tau phosphorylation was analyzed to evaluate neurodegenerative signaling. In the PDM group, p-Tau217 expression was increased compared with the PCM group, but in the PDM/TPA group, it was reduced compared with the PDM group.

**Figure 4:**
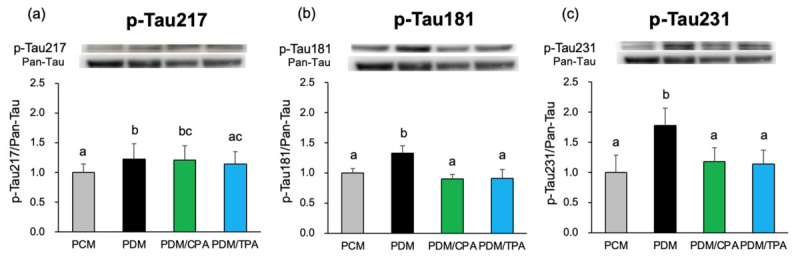
Alterations in tau phosphorylation in offspring brains. Western blot analysis of tau phosphorylation in brain tissue. Representative immunoblots of phosphorylated Tau (p-Tau217, p-Tau181, p-Tau231) and total Tau (Pan-Tau). (**a**–**c**) Quantification of phosphorylated and total tau levels. Data are presented as mean ± SEM. Statistical analyses were performed using one-way analysis of variance (ANOVA), followed by Tukey’s honest significant difference post hoc test. Different letters (a–c) indicate statistically significant differences between groups (*p* < 0.05).

The expression of p-Tau181 was higher in the PDM group than in the PCM group and lower in the PDM/CPA and PDM/TPA groups than in the PDM group. Similarly, p-Tau231 expression was increased in the PDM group and decreased in the PDM/CPA and PDM/TPA groups. Pan-tau expression was also higher in the PDM group than in the PCM group, and lower in the PDM/CPA and PDM/TPA groups than in the PDM group.

### 3.5. Gene Expression Changes in the Brain

To evaluate the effects of maternal hyperglycemia on the offspring’s cerebrum, mRNA expression levels of genes involved in neurotransmission, inflammation, and oxidative stress were analyzed.

#### 3.5.1. Inflammation-Related Genes (Figure 5)

In the analysis of inflammation-related genes, mRNA expression levels of *IL-6* and *TNF-α* were higher in the PDM group than in the PCM group. Similarly, RAGE expression, which encodes the receptor for AGEs, increased in the PDM group.

**Figure 5:**
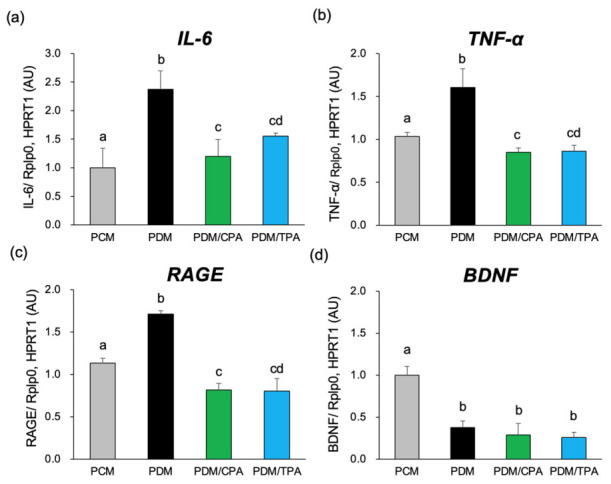
Increased inflammatory gene expression and decreased *BDNF* in PDM offspring. The mRNA expression levels of inflammatory cytokines and neurotrophic factors in the brain tissues were measured using RT-qPCR. (**a**–**c**) Relative mRNA expression of *IL-6*, *TNF-α*, and *RAGE*. (**d**) Relative mRNA expression of *BDNF*. Gene expression levels were normalized to those of housekeeping genes and expressed relative to PCM. Data are presented as mean ± SEM. Statistical analyses were performed using one-way analysis of variance (ANOVA), followed by Tukey’s honest significant difference post hoc test. Different letters (a–d) indicate statistically significant differences between groups (*p* < 0.05).

In contrast, the expression of these inflammation-related genes was lower in the PDM/CPA and PDM/TPA groups than in the PDM group. The expression of *BDNF*, a neurotrophic factor, was lower in the PDM group than in the PCM group. *BDNF* expression was lower in the PDM/CPA and PDM/TPA groups than in the PCM group.

#### 3.5.2. Neurotransmission-Related Genes (Figure 6)

**Figure 6:**
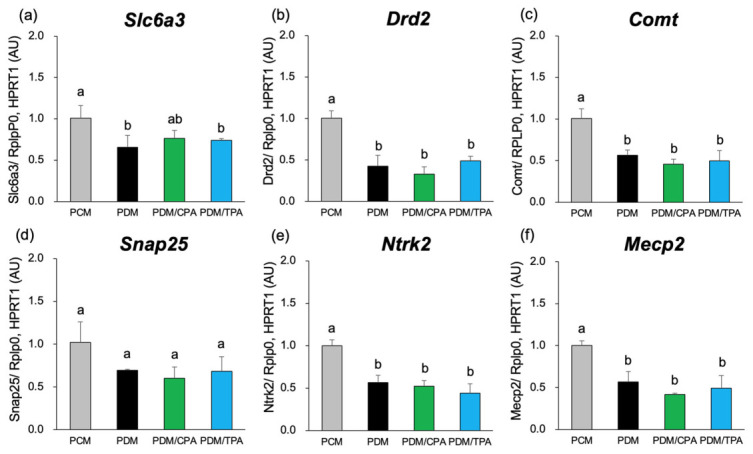
Altered expression of neuronal function-related genes in PDM offspring. mRNA expression levels of genes associated with neuronal function in the brain tissue. (**a**–**f**) Relative mRNA expression of *Slc6a3*, *Drd2*, *Comt*, *Snap25*, *Ntrk2*, and *Mecp2*. Gene expression levels were normalized to those of housekeeping genes and expressed relative to PCM. Data are presented as mean ± SEM. Statistical analyses were performed using one-way analysis of variance (ANOVA), followed by Tukey’s honest significant difference post hoc test. Different letters (a,b) indicate statistically significant differences between groups (*p* < 0.05).

Analysis of neurotransmission-related genes revealed that the expression levels of *Slc6a3*, *Drd2*, and *Comt* were lower in the PDM group than in the PCM group. No significant changes in gene expression were observed in the PDM/CPA and PDM/TPA groups compared with the PDM group. The expression of *Snap25*, a synapse-related gene, was also tended to decrease in the PDM group compared with that in the PCM group, with no significant changes observed in the PDM/CPA and PDM/TPA groups (Figure 6).

#### 3.5.3. Neurodevelopment-Related Genes (Figure 6)

Analysis of neurodevelopment-related genes revealed that Ntrk2 and Mecp2 expression levels were lower in the PDM group than in the PCM group. No significant differences in gene expression were observed between the PDM/CPA and PDM/TPA groups (Figure 6).

#### 3.5.4. Neuronal Markers Genes (Figure 7)

The expression levels of *Map2* and *Rbfox3* (NeuN) in the PDM group were lower than those in the PCM group. No difference in *Tubb3* expression levels was observed between the groups.

**Figure 7:**
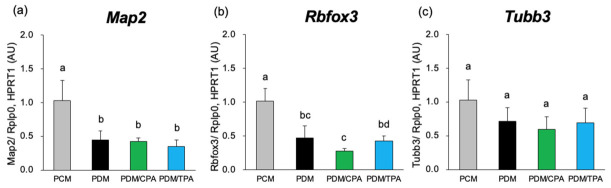
Expression of neuronal marker genes in the brains of aged offspring rats. Relative mRNA expression levels of neuronal marker genes in brain tissues from aged offspring rats were analyzed by RT-qPCR. (**a**–**c**) Relative mRNA expression levels of *Map2*, *Rbfox3*, and *Tubb3* were normalized to housekeeping genes (Rplp0 and HPRT1) and expressed relative to the PCM group. Data are presented as mean ± SEM (*n* = 5 per group). Statistical analyses were performed using one-way ANOVA followed by Tukey’s HSD post hoc test. Different letters (a–d) indicate statistically significant differences between groups (*p* < 0.05).

#### 3.5.5. Oxidative Stress-Related Genes (Figure 8)

**Figure 8:**
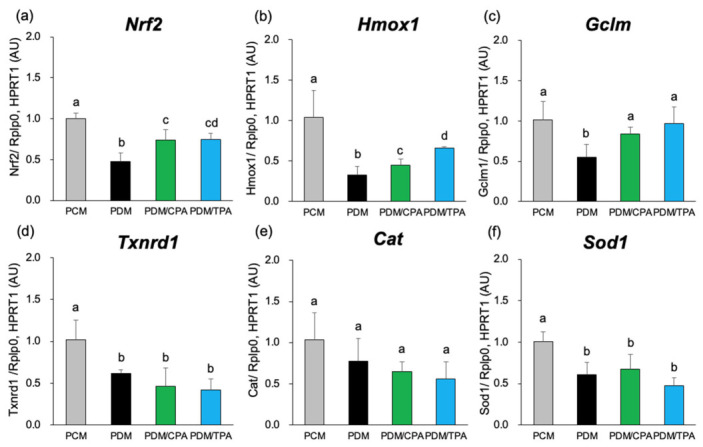
Downregulation of antioxidant-related genes in PDM offspring. The relative mRNA expression levels of antioxidant-related genes associated with the Nrf2 antioxidant pathway were analyzed in the brain tissue of aged offspring. (**a**–**f**) Relative mRNA expression levels of Nrf2, Hmox1, Gclm, Txnrd1, Cat, and Sod1 were determined by RT-qPCR. Gene expression levels were normalized to housekeeping genes and expressed relative to the PCM group. Data are presented as mean ± SEM (*n* = 5 per group). Statistical analyses were performed using one-way analysis of variance (ANOVA), followed by Tukey’s honest significant difference post hoc test. Different letters (a–d) indicate statistically significant differences between groups (*p* < 0.05).

Analysis of antioxidant-related genes revealed that *Nrf2* expression was lower in the PDM group than in the PCM group. In the PDM/CPA and PDM/TPA groups, *Nrf2* expression was higher than that in the PDM group (Figure 8). The expression levels of *Hmox1* and *Gclm* decreased in the PDM group and increased in the PDM/CPA and PDM/TPA groups compared to those in the PDM group. In contrast, the expression levels of *Txnrd1* and *Sod1* decreased in the PDM group, but no significant changes were observed in the PDM/CPA and PDM/TPA groups compared with the PDM group. No marked differences in the expression of *Cat* were observed among the groups.

### 3.6. Behavioral Alterations in Offspring Exposed to Maternal Hyperglycemia

Behavioral analyses were performed on 48-week-old male offspring to evaluate the long-term effects of fetal hyperglycemic exposure on cognitive and emotional functions.

#### 3.6.1. Y-Maze Test (Figure 9)

**Figure 9:**
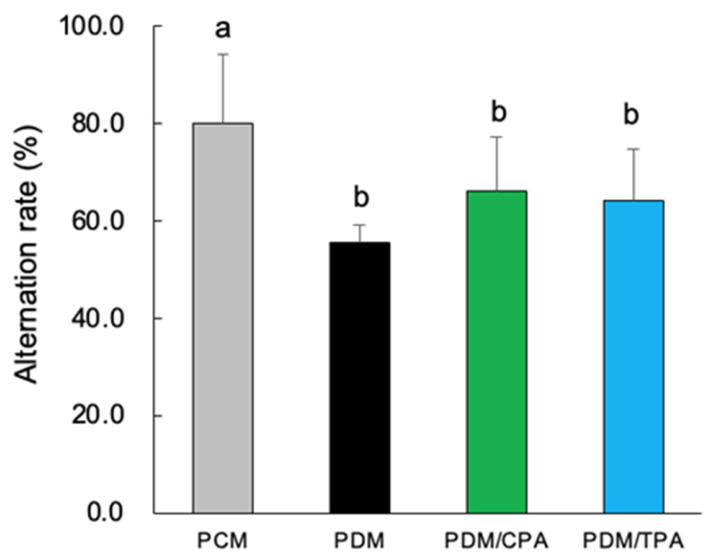
Impaired spatial working memory in PDM offspring assessed using the Y-maze test. Spontaneous alternation behavior in the Y-maze was used to evaluate spatial working memory in the aged offspring of normoglycemic mothers (PCM), diabetic mothers (PDM), and diabetic mothers treated with CPA or TPA (PDM/CPA, PDM/TPA). Spontaneous alternation rates (%) were calculated as an index of working memory performance. Data are presented as mean ± SEM (*n* = 5). Statistical analyses were performed using Welch’s ANOVA, followed by Games–Howell post hoc testing owing to unequal variances among groups. Different letters (a,b) indicate statistically significant differences between groups (*p* < 0.05). A tendency toward reduced spontaneous alternation behavior was observed in the PDM group compared to the PCM group.

The Y-maze test was used to evaluate the spatial working memory. The percentage of spontaneous alteration was significantly lower in the PDM group than in the PCM group (Figure 9). In contrast, the PDM/CPA and PDM/TPA groups showed a tendency toward increased spontaneous alternation compared to the PDM group, although the differences were not statistically significant. In the Y-maze test, the PDM group showed a tendency toward reduced spontaneous alternation compared to the PCM group, suggesting impaired spatial working memory; however, post hoc analysis did not reach statistical significance after correction for unequal variances.

#### 3.6.2. Elevated Plus Maze Test (Figure 10)

**Figure 10:**
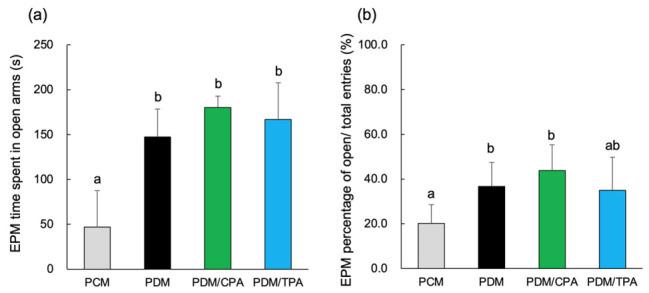
Altered anxiety-like behavior in PDM offspring assessed using the elevated plus maze. Behavioral performance in the elevated plus maze (EPM) test. (**a**) Time spent in the open arms during a 300 s test session. (**b**) Percentage of open-arm entries relative to the total arm entries. Data are presented as mean ± SEM (*n* = 5). Statistical analyses were performed using one-way ANOVA, followed by Tukey’s HSD post hoc test after confirmation of the normality and homogeneity of variance. Different letters (a,b) indicate statistically significant differences between groups (*p* < 0.05).

In the elevated plus maze test, the time spent in the open arms was higher in the PDM group than in the PCM group (Figure 10a). In addition, the percentage of open-arm entries relative to the total number of entries increased in the PDM group (Figure 10b). No significant differences in these parameters were observed between the PDM/CPA and PDM/TPA groups and the PDM group.

#### 3.6.3. Novel Object Recognition Test (Figure 11)

**Figure 11:**
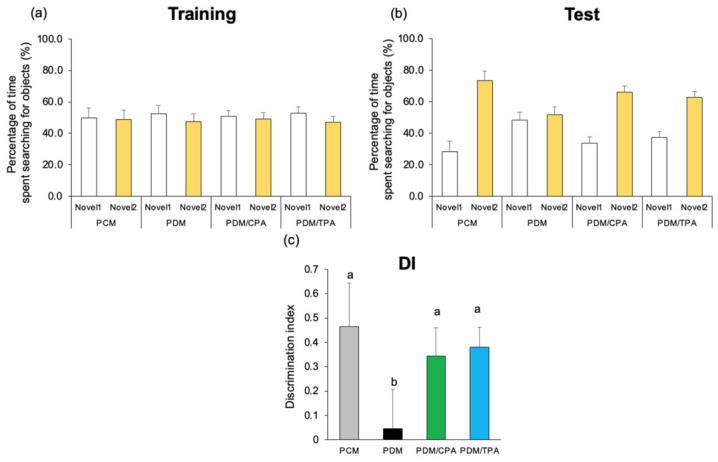
Impaired recognition memory in PDM offspring assessed using the novel object recognition test. A novel object recognition (NOR) test was performed to evaluate recognition memory. (**a**) Training session: exploration time for two identical objects (Novels 1 and 2). (**b**) Test session: exploration time for familiar and novel objects. (**c**) The discrimination index (DI) is calculated as a measure of recognition memory. Data are presented as mean ± SEM (*n* = 5). Statistical analyses were performed using one-way ANOVA, followed by Tukey’s HSD post hoc test after confirmation of the normality and homogeneity of variance. Different letters (a,b) indicate statistically significant differences between groups (*p* < 0.05).

Recognition memory was evaluated using the novel object recognition test. During the training session, no differences in exploration time for the two objects were observed between the groups (Figure 11a). During the test session, the PCM, PDM/CPA, and PDM/TPA groups spent more time exploring the novel than the familiar object, whereas no clear difference was observed between the two objects in the PDM group (Figure 11b).

The discrimination index (DI) was significantly lower in the PDM group than in the PCM group (Figure 11c). In the PDM/CPA and PDM/TPA groups, the DI was higher than that in the PDM group.

## 4. Discussion

The present study suggests that maternal hyperglycemia may induce persistent activation of the AGE–RAGE signaling pathway and disruption of Nrf2-dependent antioxidant defense mechanisms in the offspring’s brain, resulting in neuroinflammation, impaired insulin signaling, tau phosphorylation, neural dysfunction, and cognitive decline. Specifically, we evaluated aged (48-week-old) male offspring to provide insights into the long-term effects of fetal hyperglycemic exposure on brain aging. This is particularly important in the context of the Developmental Origins of Health and Disease, as many previous studies have focused on early-life outcomes rather than late-life neurological functions.

The OGTT results showed no apparent impairment of glucose tolerance in the adult offspring. This finding suggests that the effects of gestational diabetes do not necessarily persist as systemic metabolic abnormalities but may instead exert selective and long-lasting effects on the central nervous system. Such “latent central abnormalities” may reflect developmental programming induced by the fetal environment [23,24].

Furthermore, the present study confirmed increased expression of AGEs and RAGE in the PDM group, which is consistent with previous reports showing increased RAGE expression in the fetal brain in gestational diabetes models [25]. RAGE activation has been suggested to induce inflammation and oxidative stress through NF-κB signaling and function upstream of neural dysfunction [25]. Additionally, increased RAGE expression is associated with enhanced hippocampal excitability and behavioral abnormalities [9,25], which is consistent with the present findings.

Importantly, p-Akt473, an indicator of insulin signaling, was markedly decreased in the PDM group. The PI3K/Akt pathway is a major insulin signaling pathway in the brain, and its impairment contributes to cognitive decline and neurodegeneration-related molecular changes such as “brain insulin resistance” [26]. Indeed, changes in insulin receptors and IGF-1 signaling in fetal and neonatal hippocampi have been reported in gestational diabetes models [27], suggesting that impaired insulin signaling may be programmed during the fetal period and contribute to increased susceptibility to age-related neural dysfunction later in life [10,17].

Moreover, it has been reported that diabetes and hyperglycemia promote Tau phosphorylation through impairment of the PI3K/Akt pathway, leading to neurodegeneration-related molecular changes and cognitive decline [28,29]. Recent evidence supports an association between diabetes-related metabolic dysfunction, tau phosphorylation, and accelerated cognitive decline [29]. Therefore, the reduction in p-Akt levels observed in the present study may serve as a molecular basis for subsequent tau phosphorylation and neural dysfunction.

Although reduced Akt phosphorylation and increased Tau phosphorylation were observed, direct assessments of insulin receptor signaling, GSK-3β activity, or neuronal degeneration were not performed in the present study. Therefore, these molecular alterations should be interpreted as indirect evidence of impaired insulin-related signaling and neurodegeneration-related pathways, rather than definitive proof of brain insulin resistance or neurodegeneration.

A particularly important finding of this study was that despite enhanced oxidative and glycation stress, the expression of *Nrf2* and its downstream antioxidant genes was reduced. This “failure of antioxidant responses in the presence of stress” suggests not merely insufficient compensation but also dysfunction of the Nrf2 pathway itself, driven by chronic AGE–RAGE signaling and aging [18,30]. Recent studies have highlighted the importance of Nrf2 dysfunction and mitochondrial oxidative stress in age-related molecular changes underlying neurodegeneration [30].

Several mechanisms may underlie this impaired *Nrf2* response: (1) age-related decline in the *Nrf2/ARE* system, (2) chronic inflammation mediated by AGE–RAGE signaling, which may favor NF-κB signaling and suppress *Nrf2*-dependent antioxidant responses, and (3) abnormalities in intracellular signaling, represented by decreased p-Akt, which may inhibit the upstream activation of *Nrf2* [21,31,32].

Additionally, analysis of molecules related to neural function revealed increased tau phosphorylation and decreased expression of *BDNF*, *Slc6a3*, *Drd2*, *Ntrk2*, and *Mecp2* in the PDM group. These changes suggest that glycation stress and inflammation extend to neural dysfunction [18,19,33], and that reduced Akt signaling and disruption of *Nrf2*-dependent defense mechanisms may contribute to neurodegeneration-related molecular changes. These findings suggest that impairment of Nrf2-dependent antioxidant defenses and decreased Akt signaling may disrupt neural plasticity and synaptic function, ultimately leading to cognitive decline and neurodegeneration-related molecular changes [18].

Furthermore, the increased expression of GFAP and Iba1 increases neuroinflammation. Previous studies using animal models of gestational diabetes have also reported glial activation and increased levels of inflammatory cytokines in the hippocampus [10,14,34], which are consistent with the present findings. Excessive microglial activation contributes to abnormal neural circuit formation and cognitive dysfunction [35], suggesting that the neuroinflammation observed in this study may represent an important pathological basis for neural circuit dysfunction and cognitive impairment in offspring exposed to maternal hyperglycemia [10,35]. Recent reviews have also emphasized persistent microglial activation and neuroinflammation as key mechanisms linking gestational diabetes exposure to long-term neuropsychiatric abnormalities in the offspring [26].

Although no marked changes were observed in the synapse-related proteins PSD-95 and synaptophysin [36], decreases in these proteins have been reported in models of diabetes and Alzheimer’s disease [37], suggesting that there may be differences depending on the brain region or disease stage. The increase in NeuN protein expression does not necessarily directly reflect an increase in neuronal number but may instead indicate altered neuronal status or dysregulated neuronal expression [38]. Supporting this interpretation, additional RT-qPCR analyses revealed decreased expression of neuronal markers, including Map2 and Rbfox3 (NeuN), in the PDM group, whereas Tubb3 expression was relatively preserved (Figure 7). These observations indicate that maternal hyperglycemia may induce alterations in neuronal phenotype or integrity rather than simple neuronal proliferation [38].

Interestingly, despite substantial alterations in inflammatory signaling, Tau phosphorylation, and antioxidant-related pathways, synaptic markers such as PSD-95 and synaptophysin showed relatively limited changes in the present study. These findings may suggest that the observed molecular abnormalities reflect early or partial neurodegeneration-related processes rather than severe synaptic degeneration. Alternatively, synaptic alterations may occur in a brain region-specific or temporally restricted manner that was not fully captured in the present analyses.

Moreover, increased tau phosphorylation (p-Tau217, p-Tau181, and p-Tau231) indicated enhanced signaling in neurodegeneration-related molecular changes, consistent with previous reports of tau abnormalities associated with diabetes and insulin resistance [17,39,40]. In particular, decreased p-Akt is known to promote Tau phosphorylation through activation of GSK-3β [41], while AGE–RAGE signaling can also enhance Tau phosphorylation via inflammation and oxidative stress [20]. Therefore, the present findings support a molecular mechanism by which AGE–RAGE signaling induces inflammation and insulin resistance, thereby promoting tau phosphorylation. Emerging evidence suggests a bidirectional relationship between insulin resistance and tau pathology, forming a vicious cycle that promotes neurodegenerative molecular changes [42].

In contrast, although the effects of CPA and TPA on AGE–RAGE signaling were limited, recovery of p-Akt, reduction in inflammatory cytokines, and partial restoration of *Nrf2*-related genes were observed. These findings suggest that palmitoleic acid primarily acts on insulin signaling and anti-inflammatory/antioxidant pathways, thereby attenuating the negative effects of “metabolic memory.” Palmitoleic acid is recognized as a lipokine with insulin-sensitizing and anti-inflammatory properties [43], which is associated with our results.

In the Y-maze test, the PDM group showed a reduced spontaneous alternation rate, suggesting an impaired spatial working memory. Furthermore, a decreased discrimination index was observed in a novel object recognition test, indicating impaired recognition memory. These observations suggest that fetal exposure to hyperglycemia may have long-lasting effects on memory. Previous studies on the offspring of gestational diabetes model animals have reported hippocampus-dependent memory impairment, particularly reduced learning and memory performance in the Y-maze and Morris Water Maze tests [9,11]. The present findings are consistent with these reports and suggest that fetal hyperglycemic exposure may affect hippocampal function.

Furthermore, the reduction in *BDNF* levels and the suppression of the *Nrf2* pathway observed in this study may contribute to memory impairment by decreasing synaptic plasticity and impairing neuroprotective mechanisms [8,44]. Increased Tau phosphorylation is closely associated with synaptic dysfunction and may represent the molecular basis of impaired recognition memory [45]. The novel object recognition test reflects the function of the hippocampus and entorhinal cortex. Impairments in this test are associated with neurodegeneration-related molecular changes and synaptic dysfunction [46]. Similar memory deficits have been reported in gestational diabetes models [11,47,48], which are consistent with the present findings.

In contrast, in the elevated plus maze test, the PDM group spent more time on the open arms and had a higher percentage of open-arm entries. These changes may reflect altered emotional or exploratory behavior. However, increased open-arm exploration in the elevated plus maze can be influenced by multiple behavioral dimensions, including reduced anxiety-like behavior, disinhibition, exploratory drive, and locomotor activity. Because locomotor activity was not independently quantified, these findings should be interpreted cautiously. Previous studies using gestational diabetes models have reported altered anxiety-related behavior and increased activity [9,10,11,48,49], which may be associated with Attention-Deficit/Hyperactivity Disorder-like behavior. Additionally, epidemiological studies have reported an increased risk of ADHD in children born to mothers with diabetes [1,3]. Therefore, the behavioral findings of the present study are consistent with those of previous epidemiological and developmental studies in humans [50]. A recent large-scale synthesis study reported increased risks of ADHD and autism spectrum disorders in children with gestational diabetes in utero.

These behavioral changes are likely closely related to the molecular abnormalities observed in this study. In the PDM group, enhanced AGE–RAGE signaling and increased inflammatory cytokine levels were accompanied by decreased Akt signaling, reduced *BDNF* expression, and downregulation of *Nrf2*-related genes. These changes may contribute to cognitive impairment by disrupting neural plasticity, synaptic integrity, and neuronal signaling, and enhanced tau phosphorylation is considered an important factor in neural dysfunction and cognitive decline associated with neurodegenerative processes [40,45,51,52].

Abnormalities in the dopaminergic system may also contribute to the observed behavioral changes. In the present study, reduced expression of *Slc6a3* and *Drd2* was observed, suggesting impaired dopaminergic neurotransmission. The dopamine system plays a critical role in motor control and attention, and its dysfunction is associated with hyperactivity and impulsivity [53]. Therefore, the hyperactivity-like behavior observed in this study may be closely related to impaired dopaminergic neurotransmission and reward-related signaling, particularly the dysfunction of dopamine transporters and dopamine receptor pathways associated with attention and motor regulation [53,54].

Although the observed molecular alterations are biologically consistent with previous reports on neuroinflammatory and neurodegeneration-related pathways, the present study did not provide direct mechanistic evidence linking AGE–RAGE signaling, Akt suppression, Nrf2 dysfunction, tau phosphorylation, and behavioral abnormalities. Therefore, these relationships should be interpreted as hypothetical or associative mechanisms requiring further experimental validation.

In contrast, the CPA- and TPA-treated groups showed a tendency toward partial attenuation of molecular and behavioral alterations in both the Y-maze and novel object recognition tests. These changes may be associated with reduced RAGE expression, reduced inflammation, and the partial recovery of Akt signaling and antioxidant responses. Palmitoleic acid is known to improve insulin sensitivity and exert anti-inflammatory effects [55], which are associated with p-Akt and the reduction in inflammatory gene expression observed in this study. In particular, restoration of brain insulin signaling has been reported to improve synaptic plasticity and memory function [17].

However, the behavioral improvements induced by CPA and TPA were only partial, and no significant recovery was observed in any parameter. This finding suggests that neurodevelopmental abnormalities induced by fetal hyperglycemia, such as reduced *BDNF* and *Mecp2* expression, may persist irreversibly.

Although CPA and TPA supplementation partially attenuated several molecular and behavioral abnormalities, some behavioral improvements were not statistically significant. Therefore, the protective effects of palmitoleic acid should be interpreted with caution. Additional studies using larger cohorts and more comprehensive behavioral analyses are necessary to confirm these effects.

The CPA and TPA doses used in the present study were selected based on previous experimental studies that demonstrated their biological activity in maternal hyperglycemia models. However, the translational relevance of these supplementation protocols for human nutritional intake remains unclear. Although palmitoleic acids are naturally occurring dietary fatty acids present in dairy products and certain plant oils, the doses administered in the present animal study may have exceeded typical human dietary exposure. Therefore, the present findings should be interpreted as proof-of-concept evidence, and future studies are required to determine physiologically relevant intake ranges, long-term safety, and clinical applicability in humans.

Although these molecular changes appear to be associated with the observed behavioral abnormalities, further studies are needed to address the limitations of this study.

Several limitations of the present study should be acknowledged. First, the STZ-induced maternal hyperglycemia model used in this study does not fully replicate the pathophysiology of classical human gestational diabetes mellitus, particularly insulin resistance-driven metabolic abnormalities characteristic of human GDM. Because STZ administration at gestational day 2 induces hyperglycemia from early pregnancy, this model may reflect a relatively severe maternal hyperglycemic condition rather than late-onset human GDM. Second, only male offspring were analyzed in order to reduce variability associated with estrous cycle-related hormonal fluctuations and to facilitate stable long-term behavioral analyses; therefore, potential sex-specific responses could not be evaluated. In addition, although molecular alterations associated with AGE–RAGE signaling, impaired Akt phosphorylation, Nrf2-related pathways, and Tau phosphorylation were observed, direct mechanistic relationships among these pathways and behavioral abnormalities were not established. Oxidative stress and inflammatory responses were evaluated primarily using gene expression and signaling-related analyses without direct biochemical measurements of ROS production, cytokine protein levels, or antioxidant enzyme activities. Furthermore, behavioral analyses were performed using manual scoring rather than automated tracking systems, and locomotor activity was not independently evaluated using an open field test. Therefore, the elevated plus maze findings should be interpreted cautiously because altered open-arm behavior may reflect multiple behavioral dimensions, including anxiety-related behavior, exploratory activity, disinhibition, or locomotor alterations. Collectively, these limitations indicate that the present findings should be interpreted as exploratory and hypothesis-generating rather than definitive mechanistic evidence.

Taken together, maternal hyperglycemia appears to induce persistent activation of the AGE–RAGE axis and disruption of *Nrf2*-dependent antioxidant defense mechanisms by altering the fetal metabolic environment. This state of “inflammation dominance and impaired defense” may subsequently progress to neural dysfunction through impaired insulin signaling and enhanced Tau phosphorylation. The present study demonstrates that the fetal metabolic environment can exert long-term effects on brain function later in life and suggests that interventions targeting *Nrf2* may represent a promising therapeutic strategy. Therefore, the present findings should be interpreted as exploratory and hypothesis-generating rather than as definitive mechanistic evidence.

## 5. Conclusions

The present study suggests that maternal hyperglycemia is associated with long-lasting alterations in molecular signaling and cognitive function in aged male offspring, potentially involving persistent activation of AGE–RAGE signaling, impaired insulin signaling, disruption of Nrf2-dependent antioxidant defense pathways, neuroinflammation, and enhanced tau phosphorylation. Maternal palmitoleic acid supplementation partially ameliorated several of these molecular and behavioral abnormalities, including inflammatory signaling and antioxidant responses.

These findings further suggest that an intrauterine hyperglycemic environment may contribute to increased vulnerability to age-related neurological dysfunction later in life, supporting the concept of developmental programming associated with maternal metabolic status. However, because the present study used an experimental animal model and did not establish direct causal relationships between the observed molecular alterations and behavioral phenotypes, the findings should be interpreted cautiously.

This study had several limitations. First, the STZ-induced maternal hyperglycemia model does not fully reproduce the pathophysiology of human gestational diabetes mellitus, particularly the insulin resistance-driven mechanisms. Second, only male offspring were analyzed; therefore, potential sex-specific differences could not be evaluated. Third, behavioral analyses were performed manually, which may have introduced observer bias despite efforts to standardize procedures. In addition, although multiple molecular pathways were investigated, mechanistic interventions targeting AGE–RAGE, Nrf2, or insulin signaling were not performed, limiting conclusions regarding causality. Furthermore, detailed brain region-specific analyses and histopathological evaluations were not performed.

Future studies using larger cohorts and experimental models that more closely replicate human gestational diabetes, particularly diet-induced insulin resistance models, will be important for validating and extending the present findings. Inclusion of both male and female offspring may further clarify sex-specific differences in neurodevelopmental and neurodegenerative outcomes. In addition, mechanistic studies targeting AGE–RAGE, Nrf2, and insulin-related pathways, together with region-specific brain analyses, histopathological evaluation, and advanced neuroimaging approaches, may help clarify the causal mechanisms linking maternal hyperglycemia to late-life neurological dysfunction. Longitudinal investigations tracking the progression from early metabolic alterations to age-related cognitive decline may further improve understanding of developmental programming mechanisms and potential preventive nutritional interventions.

## Data Availability

All data generated or analyzed during this study are included in this published article.

## Supplementary Materials

The following supporting information can be downloaded at: https://www.mdpi.com/article/10.3390/nu18111748/s1, Table S1: Primer sequences used in this study.

## Abbreviations

The following abbreviations are used in this manuscript:

GDM	Gestational diabetes mellitus
CPA	Cis-palmitoleic acid
TPA	Trans-palmitoleic acid
PCM	Offspring of control dams
PDM	Offspring of hyperglycemic dams
AGEs	Advanced glycation end products
RAGE	Receptor for advanced glycation end products
NF-κB	Nuclear factor kappa B
GFAP	Glial fibrillary acidic protein
Iba1	Ionized calcium-binding adapter molecule 1
RT-qPCR	Reverse transcription quantitative polymerase chain reaction
DI	Discrimination index
SEM	Standard error of the mean
ANOVA	Analysis of variance
OGTT	Oral glucose tolerance test
AUC	Area under the curve
p-Akt473	Phosphorylated Akt at Ser473
Akt	Protein kinase B
PSD-95	Postsynaptic density protein 95
NeuN	Neuronal nuclei
IL-6	Interleukin-6
TNF-α	Tumor necrosis factor-alpha
BDNF	Brain-derived neurotrophic factor
Slc6a3	Solute carrier family 6 member 3
Drd2	Dopamine receptor D2
Comt	Catechol-O-methyltransferase
Snap25	Synaptosomal-associated protein 25
Ntrk2	Neurotrophic receptor tyrosine kinase 2
Mecp2	Methyl-CpG-binding protein 2
Nrf2	Nuclear factor erythroid 2-related factor 2
Hmox1	Heme oxygenase 1
Gclm	Glutamate-cysteine ligase modifier subunit
Txnrd1	Thioredoxin reductase 1
Cat	Catalase
Sod1	Superoxide dismutase 1
EPM	Elevated plus maze
NOR	Novel object recognition
IGF-1	Insulin-like growth factor-1
PI3K	Phosphoinositide 3-kinase
GSK-3β	Glycogen synthase kinase-3 beta
