# Supplementary material for: Maternal Hyperglycemia Promotes Late-Life Neurodegenerative Changes Associated with AGE–RAGE Activation and Impaired Insulin Signaling: Protective Effects of Palmitoleic Acid
Source: Nutrients. 2026 May 29;18(11):1748. doi: 10.3390/nu18111748 (PMC13258400; doi:10.3390/nu18111748)
Supplement: Supplementary file 1 [file nutrients-18-01748-s001.zip › nutrients-4315050-supplementary.pdf]

Supplementary TableS1 Primer sequences used in this study.

	Forward primer	Reverse primer
Rplp0	CACTGGCTGAAAAGGTCAAGG	GTGTGAGGGGCTTAGTCGAA
HPRT1	GACCGGTTCTGTCATGTCG	ACCTGGTTCATCATCACTAATCAC
IL-6	CCAGTTGCCTTCTTGGGACT	TGCCATTGCACAACTCTTTTC
TNF-α	GAGAGATTGGCTGCTGGAAC	TGGAGACCATGATGACCGTA
RAGE	AGCTTCAGTCTGGGCCTTC	CAGCTGAATGCCCTCTGG
BDNF	GAACGGGAGGGGTAGATTTC	GAGGAGGGAGGGAAAGAATG
Slc6a3	CAACTCCACCCTCATCAACC	TTTCTTGCTCCAGGTCTCCC
Drd2	CTTGAAGAGCCGTGCCACCC	TGTCTGCCTTCCCTTCTGACCC
Comt	AGAAGGAATGGGCCATGAAT	ACAGTAAGCTCCCAGCTCCA
Snap25	CCATCAGTGGTGGCTTCAT	TCAATCTCATTGCCCATGTCT
Ntrk2	GCCTGTGTATGAGAAGGGAAAG	TCACTCCTGCTGTGCTTTATG
Mecp2	ACCTTGCCTGAAGGTTGGAC	GGCTTTTCCCTGGGGATTGA
Map2	AAATCGGATCAACCGACAAC	GCCTGTGACGGATGTTCTTT
Rbfox3	AGCAGCAGCCCAAACGACTA	TTGGAGCCCCGCTCGTTAAA
Tubb3	AGCCCTCTACGACATCTGCT	ATTGAGCTGACCAGGGAATC
Nrf2	CCATTTGTAGATGACCATGAG	GTATTAAGACACTGTAACTCGG
Hmox1	CTCATCCTGAGCTGCTGGTG	GATGCTCGGGAAGGTGAAAA
Gclm	TTAGTTCAGAGCAAGAAGATTGT	TTACTATTGGGTTTTACCTGTGCC
Txnrd1	GGATTCCTGGCTGGTATCGG	TTGTGGACTTAGCGGTCACC
Cat	CAAGTTCCATTACAAGACTGAC	TTAAATGGGAAGGTTTCTGC
Sod1	GAAAGGACGGTGTGGCCAAT	CTCGTGGACCACCATAGTACG
